# Eleven-Year Trajectories of Internet Usage Time and Depression Scores Among Middle-Aged and Older Adults in China: Latent Class Mixed Model Analysis

**DOI:** 10.2196/64581

**Published:** 2025-05-26

**Authors:** Mengyao Li, Zhongliang Zhou, Jing Wang, Dan Wang, Rebecca Mitchell, Wenhua Wang

**Affiliations:** 1 School of Public Policy and Administration Xi'an Jiaotong University Xi'an China; 2 Health and Wellbeing Research Unit (HoWRU) Macquarie Business School Macquarie University Sydney Australia

**Keywords:** internet use, depressive scores, trajectory, middle-aged and older adults, latent class mixed model

## Abstract

**Background:**

Mental health issues have emerged as a global challenge, particularly affecting middle-aged and older adults. Research has shown that internet use can potentially promote mental health. Substantial research investigated the relationship between mental health and internet usage time or purposes. However, few studies have examined the association between internet usage time trajectories and mental health.

**Objective:**

The objective of this study was to identify distinct trajectories of internet usage time over a span of 11 years and assess their relationship with depressive scores among middle-aged and older adults.

**Methods:**

Using longitudinal data from the China Family Panel Studies spanning from 2010 to 2020 and consisting of 5 waves. Participants older than 45 years with internet usage data available for at least 3 waves, including wave 5, were included in the analysis. Internet usage time was operationalized as the number of hours spent on the internet per week, while depressive scores were assessed using the 8-item Center for Epidemiologic Studies Depression Scale (CES-D 8). A latent class mixed model was used to identify distinct trajectories of internet usage time over the course of this period. Mixed-effect models were used to test the relationship between distinct trajectories of internet usage time and depressive scores.

**Results:**

Among 9163 middle-aged and older adults were included in the analysis. The trajectory analysis identified 3 clusters: “Never use,” “Slow increase,” and “Rapid increase.” The “Never use” cluster indicated no internet use for one decade. In the slow increase cluster, internet use rose slowly with an average of 7.69 hours per week in 2020. In contrast, the “Rapid increase” cluster exhibited a sharp increase, reaching 15.13 hours per week in 2020. Compared to the “Never use” cluster, the “Slow increase” cluster was significantly negatively associated with depressive scores among middle-aged and older adults (coefficient –0.20, 95% CI –0.34 to –0.06), while the “Rapid increase” cluster showed no significant association. The benefits of internet use were more pronounced among females and older adults with chronic diseases than among their male and older adult counterparts without chronic diseases. The sensitive analysis confirmed the robustness of the results.

**Conclusions:**

This study identified 3 trajectory clusters of internet usage time among middle-aged and older adults in China from 2010 to 2020. Compared to the older adults who never used the internet, those whose internet usage increases gradually over time exhibited slightly lower depressive scores. Notably, the “Slow increase” cluster exhibited a negative association with depressive scores, with this association being statistically significant in females and older adults with chronic diseases, but not in males or those without chronic diseases. Future initiatives should aim to establish an older adult–friendly internet environment to facilitate internet access for older adults and promote moderate internet use.

## Introduction

Along with the development of information communication technology, the number of people using the internet is increasing rapidly. There are approximately 67% of the world’s population, 5.4 billion people, using the internet in 2023 [1]. In China, the internet users are 1079 million as of June 2023, and the internet penetration rate has grown from 34.3% in 2010 to 76.4% today [2]. Despite rapid growth rates, internet usage among older adults remains lower than that among younger adults worldwide [3]. In terms of the group not using the internet in China, 41.9% are older than 60 years and 59% are in rural areas [2]. The government of China issued opinions on promoting the development of “Internet plus Health Care” in 2018, putting forward the policy measures on health care services, public health services, medical education, science dissemination, etc [4]. To access internet health care services and meet their health care needs, individuals, especially older adults, must first be internet users. Therefore, understanding internet usage patterns, particularly trajectories, is crucial for promoting access to internet health care services.

Mental health, as a global public good, is essential to sustainable development in all countries and constitutes a fundamental human right for all individuals [5]. For depressive disorders, 5% of adults experience depression globally, particularly acute for older persons and low- and middle-income country groups [6,7]. China, a large population country with rapid aging, had a moderate prevalence rate of depression at 3.99% in 2017, and the prevalence rate decreased among individuals aged 5-54 years and increased in those over 55 years old compared to 1990 [8]. The prevalence rate of depression in individuals older than 60 years in China ranges from 11% to 57% with different measures [9]. Under the severe challenge of mental health, internet-mediated interventions could be promising solutions because they delineate the effectiveness and lower costs in changing lifestyles and noncommunicable disease management [10,11]. Digital tools play an essential role for individuals with mental disorders, facilitating access to social support, enabling the screening and diagnosis of mental disorders, and supporting treatment processes [5]. Promoting internet access and elucidating the relationship between internet use and mental health are essential for enhancing mental well-being.

Numerous studies have investigated the relationship between internet usage and mental health among older adults, yielding mixed empirical findings. Current research on internet use and mental health focuses on three main areas: first, the majority of research examines whether individuals use the internet and their frequency of use. Internet use or the frequency of use is positively associated with subjective well-being [12], psychological well-being [13], remission of loneliness [14], higher health-related quality of life [15], decreasing the risk of chronic diseases [16], and mitigating the depressive symptoms [17,18] among older adults. The benefit of the positive relationship is particularly effective for frail, low-income group, and unhealthy groups [13,19]. Noteworthy, the benefit could be offset by overuse by increasing usage frequency and length [20,21]. Nonetheless, internet use has been reported as not related to depression [22], even negatively associated with life satisfaction [23]. Second, some studies have focused on specific aspects of internet use, such as social networking sites and different purposes. Social networking site use is positively associated with life satisfaction in European older people [24]. China also has reported the same finding that using WeChat, a popular social networking site in China, is associated with lower depression and higher quality of life among older adults [25,26]. Concerning the purposes of internet use, it is divided into social (connecting with others), instrumental (banking and commercial activities), and informational (reading information) purposes [27]. The results show that internet use for entertainment and social contact is negatively related to depressive symptoms, while not relieving depression for purposes of working and commercial activity [28]. The same reported that the internet for social and leisure-related activities scored higher psychological well-being compared to the lowest frequency of internet activities [29]. Finally, some studies have focused on analyzing pathways and mechanisms underlying the relationship between internet use and mental health. Social participation and social capital are the two main factors mediating the relationship between internet use and depression, mental health, and successful aging [27,30-32].

However, longitudinal and mechanistic research in this field is scarce. The majority of studies examining the relationship between internet usage and mental health have been constrained by assessing only 2 indicators at a single time point. This cross-sectional approach fails to capture the intraindividual variability in internet use over time. Furthermore, within longitudinal studies, internet usage is expected to vary among subgroups due to the heterogeneity of older adults, who exhibit specific patterns of internet usage. For example, a cross-sectional study of internet activities among older adults showed that older adults were distinguished into four clusters: “practical users,” “minimizers,” “maximizers,” and “social users” [29]. To the best of our knowledge, there is no research on the trajectories of internet usage over longitudinal periods. Understanding the diverse patterns of internet usage among older adults sheds light on the relationship between different trajectories of internet usage and mental health outcomes.

This study uses data from 5 waves, 11 years of longitudinal data from 2010 to 2020, from The China Family Panel Studies (CFPS) to solve the questions as follows: (1) the number of distinct trajectories of internet usage time among middle-aged and older adults in China from 2010 to 2020 and (2) the relationship between distinct trajectories of internet usage time and depressive scores among Chinese middle-aged and older adults.

## Methods

### Study Design

This study was a prospective cohort study aiming to analyze the trajectories of internet usage time among middle-aged and older adults from 2010 to 2020 and to investigate the relationship between different trajectory classes of internet use and depressive scores in this population.

### Setting

All data used in this study were obtained from the CFPS, a nationally representative longitudinal survey conducted by the Institute of Social Science Survey at Peking University [33]. The baseline survey of CFPS was conducted in 2010, and the subsequent surveys were conducted every 2 years, with the latest being in 2020. The 2010 baseline survey covered 25 provinces, municipalities, or autonomous regions, using a multistage, multilevel, probabilistic sampling proportional to population size method, thereby representing 95% of the population in China [34]. With the subsequent surveys conducted, the sample size was scaled up and the survey of 2020 contained 31 provinces, municipalities, or autonomous regions. However, the key indicator of internet usage time was not surveyed in 2012. Therefore, this study used data from the 2010, 2014, 2016, 2018, and 2020 waves for analysis.

### Participants

The eligibility criteria for inclusion in this study were as follows: (1) individuals older than 45 years, as the study focused on middle-aged and older individuals and (2) individuals with data on internet usage time available from at least 3 waves of CFPS (2010, 2014, 2016, 2018, and 2020), and who were present in 2020 wave for analysis of internet usage time trajectories and depressive scores. Figure S1 in Multimedia Appendix 1 illustrates the sample selection process. Initially, there were 56,962 individuals across the 5 waves from 2010 to 2020. After excluding those younger than 45 years and those with missing internet usage time indicators, 23,192 individuals remained. Following the application of the second eligibility criterion, 9163 individuals remained, forming the sample for descriptive and regression analyses. Of these, 5134 individuals who reported 0 hours of internet usage across all waves were categorized as “never use.” The remaining 4029 individuals with recorded internet usage time were included in the trajectory analysis of internet usage. In total, 9163 individuals were analyzed to investigate the relationship between distinct internet usage trajectories and depressive scores.

### Assessments and Data Sources

#### Internet Usage Time

The internet usage time was the exposure factor that was measured at years 0, 4, 6, 8, and 10 because year 2 did not survey internet usage. At years 0, 4, 6, and 8, internet usage time was measured with one question “In general, how many hours do you use internet per week.” At year 10, internet usage time was measured “In general, how many hours do you use internet per week through mobile devices.” In the original questionnaire at year 10, internet usage was categorized as mobile device usage and computer usage independently, which caused the sum of these 2 categories over 24 hours in 1 day, because individuals can use both simultaneously. Finally, this study selected mobile device usage as internet usage time, due to the high prevalence rate of mobile devices using –99.8% of the netizens in China [2]. The internet usage time in 5 waves indicated the hours that individuals used the internet generally per week.

#### Depressive Scores

Depressive scores were the main outcome and measured with the Center for Epidemiologic Studies Depression Scale Short Form (CES-D 8), an 8-item self-reported scale of feelings or behavior that occurred in the past week [35]. The CES-D 8 was measured in 2016, 2018, and 2020, as it was not administered in the first two survey waves. Respondents answered the 8 questions using a 4-point scale ranging from “hardly ever” (less than 1 d) to “most of the time” (5-7 d), with scores ranging from 1 to 4, respectively. The total score of these 8 items serves as the indicator of the depressive scores, with higher scores signifying serious symptoms. The CES-D 8 is a well-established scale for assessing depressive scores across different ages and countries, and it has been validated in the Chinese context as well [36,37].

#### Covariates

Covariates were identified based on the literature review and previous studies [19,38,39]. Four categories of variables were considered as covariates: individual demographics (age, gender, marital status, and education level), habits and behaviors (smoking and alcohol consumption), personal economic and welfare status (medical insurance, employment status, residence, and per capita household income quantile), and health conditions (presence of chronic disease, self-reported health status, and life satisfaction). These covariates were controlled for reducing confounding factors to provide compelling evidence of depressive scores. Further details of the covariates are available in Multimedia Appendix 1.

#### Study Size

According to the eligibility criteria for inclusion and the flow chart of participant selection in Figure S1 in Multimedia Appendix 1. The final sample size for the trajectory analysis of internet usage time included 4029 individuals, and for the regression analysis examining the relationship between internet usage time trajectories and depressive scores, the sample size was 9163 individuals. We did not estimate the sample size before the analysis, as the public dataset provides sufficient volume for trajectory or longitudinal analyses, consistent with previous research [40,41]. Subsequently, we verified the statistical power of the mixed-effect model with the existing sample size using the R (R Core Team) package “simr” [42]. After 1000 simulations, the average power was 87.30% (95% CI 85.08%- 89.30%).

#### Data Analysis

Latent class mixed modeling (LCMM) was used to identify distinct trajectories of individual internet usage time across all included waves. LCMM was applied to analyze the class trajectories of the longitudinal indicator with individual-level random variation within each class [43]. LCMM was performed using the R “lcmm” package [44]. We conducted 5 models ranging from one-class to five-class to analyze the latent classes of internet usage time, incorporating a quadratic term for the year. The best-fitting model considers the lowest Akaike’s information criteria (AIC), lowest Bayesian information criteria (BIC), and at least 5% of study individuals in each class [43]. Then calculating the posterior probabilities of the selected model, the more closer to 1, indicating better classification [44]. Next, we described the sociodemographic characteristics of each trajectory class in the 2020 wave. Differences in characteristics between each trajectory were examined using the chi-square test and ANOVA.

In order to examine the relationship between internet usage time trajectories and depressive scores of individuals, a linear mixed-effect model was conducted from 2016 to 2020, incorporating all time points at which depressive scores were assessed. Linear mixed-effect models account for both fixed and random effects, allowing examination of within-participant and between-participant variations across repeated measurements [45]. The dependent variable was depressive scores, and the key independent variable was the different classes of internet usage time trajectories. All covariates were adjusted to reduce the confounder factors. Furthermore, heterogeneity analysis examined the relationship between depressive scores and internet usage time trajectories across four subgroups: sex (female vs male), residence (rural vs urban), possession of medical insurance (no vs yes), and chronic disease (no vs yes). Separate linear mixed-effect models were estimated for each subgroup, and interaction terms were included to assess significant differences in the effects of trajectories across these subgroups. Finally, to evaluate the potential effect of sample selection bias on the regression results, individuals present in all 5 waves were included in the sensitivity analysis. The sensitivity analysis mirrored the initial analysis, incorporating analysis of internet usage time trajectories and regression analysis examining the relationship between classes of internet usage time trajectories and depressive scores. Besides, to reflect depression symptoms rather than depression scores, a binary variable was created, with the cutoff score of 17 or higher on the CES-D 8 indicating significant depressive symptoms [37]. The mixed-effect logistic model from 2016 to 2020 was conducted to examine the effects of internet usage time trajectories on the depressive symptoms, adjusting for covariates.

### Ethical Considerations

The CFPS, as a longitudinal research project involving human participants, has received ethical review approval from the Peking University Biomedical Ethics Committee (approval number IRB00001052-14010). Informed consent is obtained from participants to ensure their rights to withdraw. Since this study involves secondary analysis using anonymized data from the CFPS project, no additional ethical approval was required after the data was obtained through the official website [46]. The data were anonymized when CFPS published the data, and the analysis does not involve new interactions with participants.

## Results

### Internet Usage Time Trajectories and Basic Description

**Figure 1:**
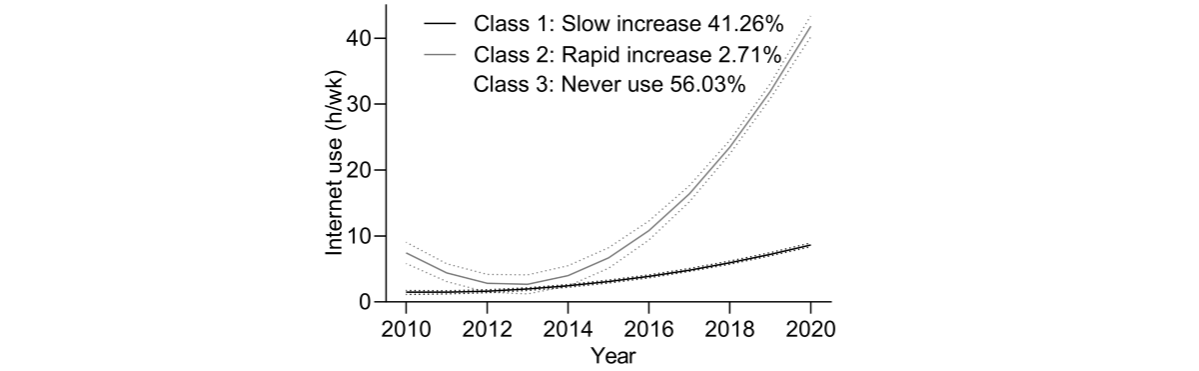
Internet use time trajectories among 9163 middle-aged and older adults. The latent class mixed model identified classes of individuals with similar trajectories of internet use over 11 years. The trajectories of internet use time were identified and showed with 95% CIs (dashed area).

Among the trajectories of the 4029 individual adults, 2 classes of trajectories were selected as the best-fitted model. All the goodness of fit indices among the 5 classes were reported in Table S1 in Multimedia Appendix. Based on the trajectory trends, 2 distinct classes were identified and labeled as “slow increase” and “rapid increase,” representing the patterns of internet usage time over a decade. Figure 1 illustrates the classes of internet usage time trajectories, with the majority, accounting for 41.26%, categorized in the slow increase group. Individuals were classified as slow increase with a mean posterior probability of 80.5% and as rapid increase with a mean posterior probability of 87.3%. Furthermore, within the cohort of 5134 individuals, internet usage time was recorded as 0, indicating that this group of individuals never used the internet. This group was designated as the “never use” class.

Among the 9163 individuals included, 1410 (10.46%) existed in 3 waves, 2555 (25.27%) existed in 2 waves, and 5198 (64.27%) existed in all 5 waves. Table 1 describes the basic characteristics of 3 classes of individuals. The “never use” class had the highest average age of 63.89 (SD 8.517), the lowest education level (93.18% below middle school), the lowest medical insurance coverage (91.34%), the lowest urban residence rate (39.43%), and the lowest per capita household income, with 67.70% in the lower quantile compared to the other classes. The “slow increase” class had the highest marriage percentage at 91.14% and the lowest percentage of individuals suffering from chronic diseases at 22.58% compared to the other classes. The depressive scores in the class of never use, slow increase, and rapid increase were 14.05, 13.19, and 13.28, respectively. In the “slow increase” cluster, internet usage time was 8.41 hours per week, whereas in the “rapid increase” cluster, it was 43.12 hours per week. In terms of the comparison among these 3 classes, the results of the chi-square test and ANOVA showed that all covariates exhibited significant differences in the 3 classes (*P*<.001), except for the tobacco consumption (*P*=.02), and alcohol consumption (*P*=.46).

**Table 1:** Characteristics of the study population in 2020.

Characteristics		Never use (n=5134)	Slow increase (n=3781)	Rapid increase (n=248)	*P* value	
Age (years), mean (SD)		63.89 (8.517)	57.48 (7.003)	57.48 (7.231)	<.001	
**Sex, n (%)**						<.001
	Female	2702 (52.63)	1742 (46.07)	128 (51.61)		
	Male	2432 (47.37)	2039 (53.93)	120 (48.39)		
**Marital status, n (%)**						<.001
	No	755 (14.71)	335 (8.86)	35 (14.11)		
	Yes	4379 (85.29)	3446 (91.14)	213 (85.89)		
**Education level, n (%)**						<.001
	Below middle school	4784 (93.18)	2684 (70.99)	130 (52.42)		
	High school and above	350 (6.82)	1097 (29.01)	118 (47.58)		
**Tobacco, n (%)**						.02
	No	3644 (72.79)	2631 (70.10)	184 (74.19)		
	Yes	1362 (27.21)	1122 (29.90)	64 (25.81)		
**Alcohol, n (%)**						.46
	No	4234 (84.58)	3138 (83.61)	210 (84.68)		
	Yes	772 (15.42)	615 (16.39)	38 (15.32)		
**Medical insurance, n (%)**						<.001
	No	428 (8.66)	229 (6.14)	11 (4.47)		
	Yes	4516 (91.34)	3502 (93.86)	235 (95.53)		
**Employment, n (%)**						<.001
	No	1783 (34.87)	1179 (31.22)	120 (48.39)		
	Yes	3331 (65.13)	2597 (68.78)	128 (51.61)		
**Residence, n (%)**						<.001
	Rural	3064 (60.57)	1545 (42.13)	41 (17.30)		
	Urban	1995 (39.43)	2122 (57.87)	196 (82.70)		
**Per capita household income quantile, n (%)**						<.001
	0%-50%	3393 (67.70)	1487 (40.04)	64 (26.45)		
	50%-100%	1619 (32.30)	2227 (59.96)	178 (73.55)		
**Chronic diseases, n (%)**						<.001
	No	3675 (73.34)	2904 (77.42)	188 (75.81)		
	Yes	1336 (26.66)	847 (22.58)	60 (24.19)		
	Health status	3.341 (1.312)	3.143 (1.176)	3.238 (1.111)	<.001	
	Life satisfaction	4.238 (0.927)	4.067 (0.908)	3.866 (0.96)	<.001	
	Depression (CES-D^a^ score)	14.05 (4.576)	13.19 (4.188)	13.28 (4.412)	<.001	
	Internet usage time	0 (0)	8.41 (7.687)	43.12 (15.13)	<.001	

^a^CES-D: Center for Epidemiologic Studies Depression Scale.

### Relationship Between Internet Usage Time Trajectories and Depressive Scores

A total of 9 covariates, including employment status, residence, chronic diseases, self-reported health status, life satisfaction, tobacco use, alcohol consumption, medical insurance, and per capita household income quantile had missing data, with a prevalence rate of less than 1.20%. Overall, 324 observations were excluded due to missing data for the dependent variable, depressive scores. Table 2 describes the relationship between classes of internet usage time trajectories and depressive scores. In comparison to the “never use” class, the “slow increase” class exhibited a negative association with the depression score (coefficient –0.20 *P*=.006), suggesting that slower increases in internet usage time were positively correlated with remission of depressive scores. However, the class of rapid increase was insignificant related to the depressive scores. Regarding the covariates, male (*P*<.001), being marital (*P*<.001), higher educational level (*P*<.001), alcohol consumption (*P*=.02), possession of medical insurance (*P*<.001), urban residence (*P*<.001), higher income (*P*<.001), and greater life satisfaction (*P*<.001) were negatively associated with the depression scores. Consuming tobacco (*P*=.008), being employed (*P*=.008), having chronic disease (*P*<.001), and having bad health status (*P*<.001) were positively correlated with depression scores. There was no significant relationship between depressive scores and age.

**Table 2:** Association between trajectories of internet use time and depressive scores.

Variables			Depressive scores, coefficient (95% CI)
**Trajectory of internet usage time (reference=never use)**			
	Slow increase	–0.20^a^ (–0.34 to –0.06)	
	Rapid increase	–0.31 (–0.70 to 0.08)	
Age (years)			–0.01 (–0.01 to 0.00)
**Sex (reference=female)**			
	Male	–1.02^b^ (–1.17 to –0.87)	
**Marital status (reference=no)**			
	Yes	–1.64^b^ (–1.83 to –1.45)	
**Education level (reference=below middle school)**			
	High school and above	–0.57^b^ (–0.74 to –0.40)	
**Tobacco (reference=no)**			
	Yes	0.20^a^ (0.05 to 0.34)	
**Alcohol (reference=no)**			
	Yes	–0.17^c^ (–0.31 to –0.03)	
**Medical insurance (reference=no)**			
	Yes	–0.45^b^ (–0.63 to –0.27)	
**Employment (reference=no)**			
	Yes	0.17^a^ (0.04 to 0.29)	
**Residence (reference=rural** **）**			
	Urban	–0.67^b^ (–0.80 to –0.55)	
**Per capita household income quantile (reference=0%-50%)**			
	50%-100%	–0.55^b^ (–0.65 to –0.44)	
**Chronic disease (reference=no)**			
	Yes	0.76^b^ (0.65 to 0.87)	
	Health status	0.71^b^ (0.67 to 0.75)	
	Life satisfaction	–0.67^b^ (–0.71 to –0.62)	

^a^*P*<.01.

^b^*P*<.001.

^c^*P*<.05.

### Heterogeneity Analysis With Different Characteristics

**Figure 2:**
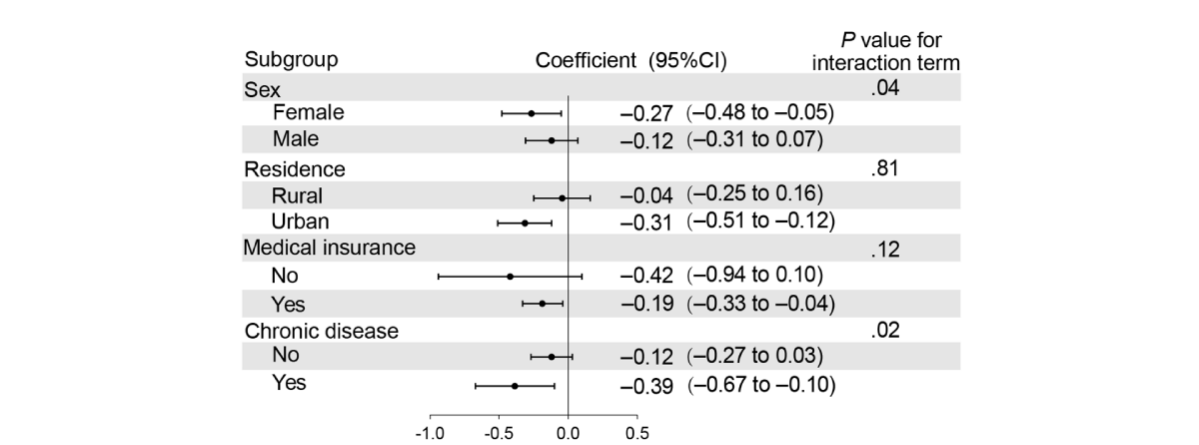
Regression results of the heterogeneity analysis. The figure shows the effect of the “slow increase” internet sage trajectory compared to “never use.” All models were adjusted for gender, marital status, education level, tobacco, alcohol, medical insurance, employment, residence, per capita household income quantile, chronic disease, health status, and life satisfaction, as outlined in Table 2.

Figure 2 shows the heterogeneity results for the relationship between internet usage time trajectories and depression scores across different subgroups. Specifically, it displays the results of “rapid increase” compared to “never use,” while the full results are available in Tables S2 and S3 in Multimedia Appendix 1. Significant subgroup differences in the effect of the “slow increase” trajectory on depression scores were observed for gender (*P*=.04 for interaction term) and chronic disease status (*P*=.02 for interaction term). The significant negative relationship between the “slow increase” class and depression scores was determined in females (coefficient –0.27, 95% CI –0.48 to –0.05), with no significant association observed in males. Among older individuals with chronic diseases, the “slow increase” class was significantly negatively associated with depression scores (coefficient –0.39, 95% CI –0.67 to –0.10), while no significant association was seen in those without chronic diseases. No significant interactions were found for residence or medical insurance, indicating that the impact of the “slow increase” in internet usage time is consistent across these subgroups.

### Sensitivity Analysis for Individuals Presenting in All Five Waves

In order to examine the robustness of the results, the individuals following all 5 waves were included as the study participants. The trajectory analysis of internet usage time and the relationship analysis between classes of internet usage time trajectories and depressive scores followed the same methods as previously reported. A total of 5198 individuals were followed up in all 5 waves. The optimal number of classes for internet usage time trajectories remained at 2. There were 1725 individuals in the “slow increase” class, while the “rapid increase” class comprised 116 individuals. Figures S2 and S3, along with Tables S4 and S5 in Multimedia Appendix 1, depict the results of the sensitivity analysis, including trajectories of internet usage time and descriptions of basic characteristics among different classes. When examining the relationship between classes of internet usage time trajectories and depressive scores in the sensitivity analysis, the “slow increase” class exhibited a negative relationship with depression scores compared to the “never use” class (coefficient –0.33, *P*=.001), whereas the “rapid increase” class showed no significant association (coefficient –0.25, *P*=.03). The relationship results were consistent with those of the primary analysis, suggesting that a gradual increase in internet usage time was significantly associated with lower depression scores among middle-aged and older adults in China.

In order to reflect the relationship between internet usage time trajectories and actual depression symptoms, the mixed-effect logistic model was used. The detailed results are provided in Table S6 in Multimedia Appendix 1. The analysis indicated that the older people in the “slow increase” class had a lower risk of depressive symptoms compared to “never use” class (odds ratio 0.87, 95% CI 0.77-0.97). No significant association was found between the “rapid increase” class and reduced risk of depressive symptoms. These results align with previous findings, suggesting that increasing the internet usage time slowly may mildly mitigate depression symptoms.

## Discussion

### Principal Findings

Using data on internet usage among middle-aged and older adults from CFPS spanning 2010-2020, this study categorized trajectories of internet usage time and investigated their relationship with depressive scores. The analysis revealed three distinct trajectories: 5134 (56.03%) middle-aged and older adults never used the internet over the decade; 3718 (41.26%) showed a slow increase in internet usage time; and 248 (2.71%) individuals exhibited a very rapid increase in internet usage time. In comparison to middle-aged and older adults who never used the internet, those with a slow increase in internet usage time exhibited a negative correlation with depressive scores, while the rapid increase group showed no significant association with depressive scores. The findings remained robust when tested on a more restricted cohort monitored over 5 waves. Replacing the dependent variable with a binary measure to reflect actual depressive symptoms yielded consistent results. Notably, among female older adults and those with chronic diseases, there was a significant relationship between a slow increase in internet usage time and depressive scores compared to their counterparts.

From 2010 to 2020 in China, 56.03% of middle-aged and older adults never used the internet; this group predominantly comprised older individuals, rural residents, and those in the lower per capita household income quantile. This distribution of internet usage was common worldwide. While the global proportion of internet users rose from 29.3% in 2010 to 53.6% in 2019 [47], significant disparities persisted across different development levels and between urban and rural areas. To address this issue, increasing the availability of internet facilities and promoting greater internet access among middle-aged and older adults is essential. On one hand, the internet infrastructure, device availability, and access fees are the prerequisites for internet access among older adults. Governments and businesses should invest in upgrading internet infrastructure to ensure service accessibility in remote areas. In addition, the development of affordable devices with basic functions tailored to older adults, supported by policy incentives to reduce purchase costs and internet fees, should be prioritized [48]. In 2023, a total of 74 of 188 economies failed to meet the affordability target for data-only mobile broadband, set at less than 2% of monthly gross national income per capita [1]. For instance, China has issued policies to promote internet health care at the governance level [4], while the Singaporean government has taken steps to improve access by renting digital devices to older adults who could not afford them [49]. On the other hand, alongside accessible and affordable internet devices, fostering digital skills and positive attitudes toward internet use among older adults is essential. Addressing concerns such as technological literacy, internet trust, and internalized ageism is vital for digital inclusion [48,50,51]. Governments and communities should take responsibility for providing training to improve digital skills among older adults [49]. Mentoring support from third parties, especially peers or family members, is also important for enhancing internet usage and skills [52,53]. In addition, enterprises should design user-friendly internet applications with simplified interfaces tailored to older adults, thereby facilitating broader access to digital services and improving overall digital integration.

Compared to middle-aged and older adults who never used the internet, those who gradually increased their internet usage exhibited a negative relationship with depressive scores. Consistent with the previous studies conducted in the United States, England, and China, internet use exhibited a significantly negative association with depressive scores among older people [17,18,54]. The physiological reasons for the result might be dopamine, which is positively correlated with weekly online time, and the role of reduced dopaminergic neurotransmission in major depression [55,56]. Besides, chatting is the dominant purpose for older people using the internet [57], which increases the frequency of contact with their children and increases the enjoyment of life [28]. In addition, another opportunity of using internet among older people is the reduction of social isolation, which is negatively associated with depressive scores [58,59]. Furthermore, receiving health information from the internet might positively modify health behavior [22,57]. Anyway, using the internet moderately has huge potential opportunities for improving the health benefits among middle-aged and older adults.

The rapid increase in internet usage time among middle-aged and older adults is not associated with depressive scores compared to nonusers. The result indicated that using the internet in moderation is important. Problematic internet use or internet addiction, defined as excessive internet usage and negative consequences such as lying, poor academic performance, and fatigue, remains primarily focused on adolescents [60-62]. Although the rapid increase in internet usage time in this study is not associated with worse mental health, the potential challenges of more rapid increases in internet usage time cannot be ignored. Previous research described that internet use was significantly associated with positive mental health or well-being, and that the benefits are counteracted by longer or more frequent internet use [20,21]. The offset effect of excessive internet use was also demonstrated in this study, although we did not know what the cutoff point of internet usage time was from benefitting mental health to nonbenefits. Avoiding rapidly increasing internet usage time and promoting reasonable internet use among middle-aged and older adults are significant for maintaining better mental health.

The effect of gradually increasing internet usage on depression scores varied significantly by gender and chronic disease status. Older adults who were female or had chronic diseases showed a stronger association between the “slow increase” trajectory and reduced depression scores, whereas this effect was not observed in their male and nonchronic disease counterparts. These findings align with existing literature, which indicates that internet use may be associated with improved mental health outcomes in females [32,63,64], this result may be due to the role of social participation in mediating the relationship between internet use and mental health. Internet use provides an accessible and cost-effective avenue for social engagement, particularly beneficial for women, who may have limited time for social interactions in China [32,63]. Regarding individuals with chronic diseases, social isolation has been shown to exacerbate morbidity and mortality [65]. Internet use offers these individuals enhanced opportunities for social contact and support, potentially improving their mental health outcomes [28]. For these vulnerable groups, promoting internet access is necessary, as they are likely to benefit significantly from it. However, findings indicate that internet usage among older females and older adults with chronic diseases remains lower than among their counterparts. This study suggests that future policies or community programs aimed at increasing internet accessibility should prioritize vulnerable older adults, particularly women and individuals with chronic diseases, by developing tailored programs that facilitate accessible and meaningful internet engagement.

### Limitations

Based on our findings, there remain 3 limitations in the analysis of internet usage among middle-aged and older adults. First, this study used internet usage time as the independent variable, failing to differentiate between various social networking sites that might be associated with depression among older adults. Second, a more detailed study focusing on the specific internet functions used by older adults should be conducted, particularly regarding health information seeking and telehealth. This study solely measured total internet usage time and did not address the specific purposes and functions of internet use among older adults. Finally, the mechanism underlying the relationship between internet usage trajectories and depressive scores warrants further investigation. Lifestyle changes, social isolation, social participation, and social capital are the possible reasons for mediating internet use and depressive scores.

### Conclusions

This study identifies three trajectories of internet usage time from 2010 to 2020 among middle-aged and older adults in China: never use, slow increase, and rapid increase. Slowly increasing the time spent on the internet and reasonably controlling its use is associated with better depressive scores among middle-aged and older adults. Furthermore, this relationship is stronger among females and individuals with chronic diseases. Future policies should focus on improving and promoting internet accessibility among middle-aged and older adults, while also promoting moderate internet usage. Subsequent research should refine crude total use time of the internet into different networking sites and functions, as well as explore the underlying mechanisms linking internet usage with depression.

## Appendix

Multimedia Appendix 1 Covariate descriptions, data cleaning flow chart, model fit indices, and results of sensitivity analyses.

## Abbreviations

AIC: Akaike’s information criteria
BIC: Bayesian information criteria
CES-D 8: Center for Epidemiologic Studies Depression Scale Short Form
CFPS: The China Family Panel Studies
LCMM: Latent class mixed model

## Data Availability

The datasets generated during and/or analyzed during this study are available from the corresponding author on reasonable request.
