# Supplementary material for: Eleven-Year Trajectories of Internet Usage Time and Depression Scores Among Middle-Aged and Older Adults in China: Latent Class Mixed Model Analysis
Source: J Med Internet Res. 2025 May 26;27:e64581. doi: 10.2196/64581 (PMC12149777; doi:10.2196/64581)
Supplement: Multimedia Appendix 1 [file jmir_v27i1e64581_app1.pdf]

Multimedia Appendix

Contents

Covariates 2
Figure S1. The flow chart of the data cleaning in the China Family Panel Survey (CFPS) 2010-2020 3
Table S1. The goodness of fit indices of all classes of trajectories 4
Table S2. Heterogeneity results of mixed effect models in different subgroups 5
Table S3 Heterogeneity results of mixed effect models with different interaction terms 7
Figure S2. The flow chart of data cleaning among individuals following up 5 waves. 9
Figure S3. Internet use time trajectories among 5,198 individuals following up 5 waves 10
Table S4. The goodness of fit indices of all classes of trajectories among individuals following up 5 waves 11
Table S5. Characteristics of the study population following up 5 waves in 2020 12
Table S6. Association between trajectories of internet use time and depressive symptoms ... 13

Covariates

Marital status was categorized into yes and no, with yes reporting married and cohabiting and no reporting unmarried, divorced, and widowed. Education level is categorized into below middle school, and high school and above. Smoking was assessed with the question "Did you smoke in the last month," and drinking with "Did you consume alcohol at least 3 times last month." Medical insurance status was determined based on whether the individual had any form of medical coverage, including urban and rural resident basic medical insurance (URRBMI), urban employee basic medical insurance (UEBMI), or other types of medical insurance. Employment status was assessed by whether individuals worked for at least 1 hour during the past week, including agricultural, wage-earning, and self-employment. The residence was measured whether the resident location of the individual was urban or rural. Presence of chronic diseases was assessed with the question "Have you been diagnosed with any chronic diseases by a doctor in the last 6 months?" Self-reported health status and life satisfaction were assessed with single questions, each using a 5-point Likert scale: "How would you rate your health status?" and "How satisfied are you with your life?" Responses ranged from 1 for very healthy/dissatisfied to 5 for very unhealthy/satisfied.

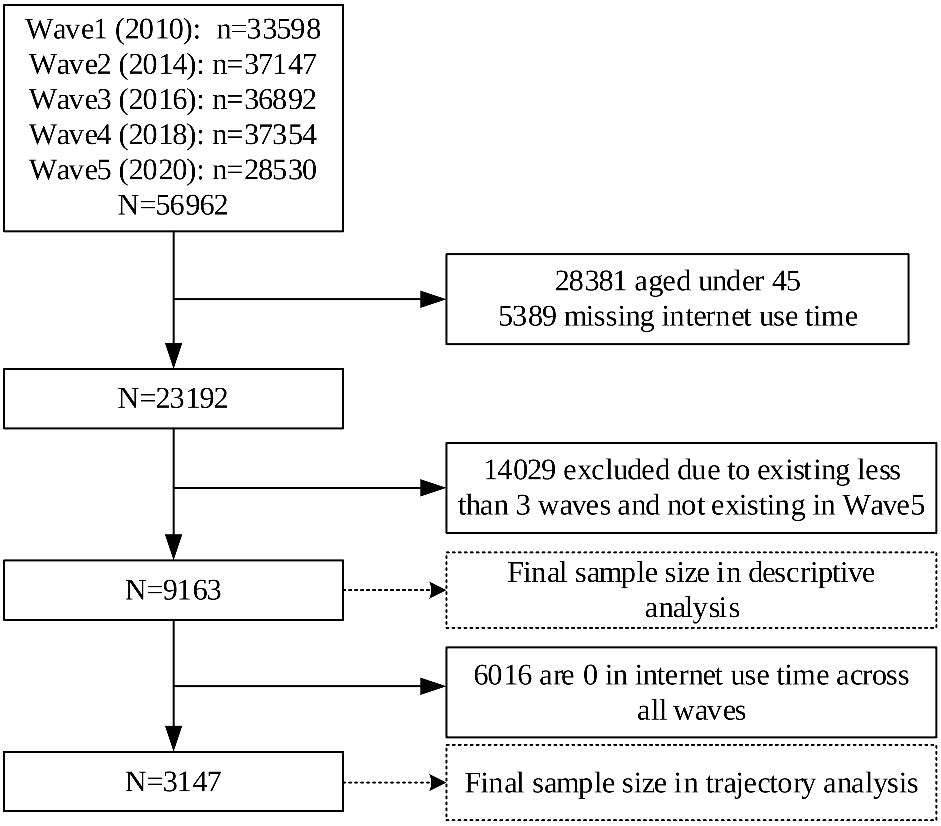

Figure S1. The flow chart of the data cleaning in the China Family Panel Survey (CFPS) 2010-2020

Table S1. The goodness of fit indices of all classes of trajectories

latent class(es)	AIC	BIC	SABIC	each class percent (%)				
				1	2	3	4	5
1	120846.6	120890.8	120868.5	100.00				
2	**119150.1**	**119219.4**	**119184.5**	93.84	6.16			
3	119158.1	119252.6	119205	93.60	0	6.40		
4	117611.3	117731	117670.6	4.54	90.67	0	4.79	
5	117619.3	117764.2	117691.1	4.86	0	89.75	5.39	0

Note: Although 4 latent classes had the lowest AIC, BIC, and SABIC, however, it did not correspond with the criterion that the minimum percent of each class should be over 5%. Accordingly, the 2 latent classes were the optimal classes of internet use time trajectories.

Table S2. Heterogeneity results of mixed effect models in different subgroups

	Female	Male	Rural	Urban	No insurance	Insurance	No chronic	Chronic
	Coef.	*Coef.*	*Coef.*	*Coef.*	*Coef.*	*Coef.*	*Coef.*	*Coef.*
	95% CI	*95% CI*	*95% CI*	*95% CI*	*95% CI*	*95% CI*	*95% CI*	*95% CI*
Trajectory of internet usage time (Ref. = Never use)								
Slowly increase	-0.27*	-0.12	-0.04	-0.31**	-0.42	-0.19*	-0.12	-0.39**
	[-0.48,-0.05]	[-0.31,0.07]	[-0.25,0.16]	[-0.51,-0.12]	[-0.94,0.10]	[-0.33,-0.04]	[-0.27,0.03]	[-0.67,-0.10]
Rapid increase	-0.36	-0.26	-0.63	-0.25	-0.59	-0.29	-0.21	-0.51
	[-0.93,0.21]	[-0.79,0.28]	[-1.51,0.25]	[-0.67,0.18]	[-2.16,0.99]	[-0.69,0.10]	[-0.63,0.21]	[-1.27,0.25]
Age	0.00	-0.01	0.01	-0.02**	-0.03	-0.01	0.00	-0.02*
	[-0.01,0.01]	[-0.02,0.00]	[-0.01,0.02]	[-0.03,-0.00]	[-0.05,0.00]	[-0.01,0.00]	[-0.01,0.01]	[-0.04,-0.00]
Gender (Ref. = Female)								
Male			-1.09***	-0.99***	-1.37***	-1.02***	-0.94***	-1.20***
			[-1.30,-0.88]	[-1.19,-0.78]	[-1.94,-0.81]	[-1.17,-0.87]	[-1.10,-0.78]	[-1.49,-0.92]
Marital status (Ref. = No)								
Yes	-1.22***	-2.24***	-1.86***	-1.40***	-1.84***	-1.62***	-1.67***	-1.59***
	[-1.48,-0.97]	[-2.53,-1.96]	[-2.13,-1.58]	[-1.66,-1.15]	[-2.43,-1.25]	[-1.82,-1.43]	[-1.88,-1.46]	[-1.94,-1.25]
Education level (Ref. = Below middle school)								
High school and above	-0.63***	-0.51***	-0.72***	-0.43***	-0.66*	-0.55***	-0.59***	-0.47**
	[-0.90,-0.35]	[-0.72,-0.30]	[-1.01,-0.44]	[-0.63,-0.23]	[-1.30,-0.01]	[-0.72,-0.38]	[-0.77,-0.41]	[-0.80,-0.13]
Tobacco (Ref. = No)								
Yes	0.41	0.15*	0.23*	0.21	0.44	0.19*	0.16*	0.34*
	[-0.01,0.84]	[0.00,0.30]	[0.03,0.43]	[-0.00,0.41]	[-0.14,1.02]	[0.05,0.34]	[0.00,0.32]	[0.03,0.65]
Alcohol (Ref. = No)								
Yes	-0.13	-0.19*	-0.24*	-0.08	0.28	-0.20**	-0.13	-0.47**
	[-0.52,0.26]	[-0.34,-0.04]	[-0.44,-0.04]	[-0.28,0.11]	[-0.31,0.86]	[-0.34,-0.05]	[-0.28,0.02]	[-0.81,-0.12]
Medical insurance (Ref. = No)								
Yes	-0.46***	-0.42**	-0.58***	-0.33**			-0.57***	-0.25

	[-0.71,-0.20]	[-0.68,-0.16]	[-0.85,-0.31]	[-0.57,-0.09]			[-0.77,-0.37]	[-0.67,0.17]
Employment (Ref. = No)								
Yes	0.19*	0.13	-0.1	0.29***	0.29	0.19**	0.29***	-0.06
	[0.02,0.36]	[-0.05,0.32]	[-0.29,0.09]	[0.13,0.45]	[-0.19,0.78]	[0.06,0.31]	[0.15,0.43]	[-0.31,0.19]
Residence (Ref. = Rural)								
Urban	-0.72***	-0.60***			-0.85***	-0.67***	-0.59***	-0.92***
	[-0.90,-0.53]	[-0.77,-0.43]			[-1.32,-0.37]	[-0.80,-0.54]	[-0.72,-0.45]	[-1.18,-0.66]
Per capita household income quantile (Ref. = 0-50%)								
50-100%	-0.61***	-0.49***	-0.47***	-0.61***	-1.01***	-0.54***	-0.54***	-0.77***
	[-0.77,-0.45]	[-0.64,-0.35]	[-0.62,-0.32]	[-0.76,-0.46]	[-1.46,-0.57]	[-0.65,-0.43]	[-0.65,-0.42]	[-1.01,-0.54]
Chronic disease (Ref. = No)								
Yes	0.87***	0.62***	0.90***	0.63***	0.51*	0.78***		
	[0.71,1.02]	[0.46,0.78]	[0.74,1.05]	[0.48,0.79]	[0.01,1.02]	[0.67,0.89]		
Health status	0.77***	0.64***	0.72***	0.70***	1.01***	0.70***	0.68***	0.89***
	[0.71,0.83]	[0.58,0.70]	[0.66,0.77]	[0.64,0.76]	[0.84,1.19]	[0.66,0.74]	[0.64,0.73]	[0.79,0.99]
Life satisfaction	-0.68***	-0.66***	-0.68***	-0.67***	-0.76***	-0.68***	-0.67***	-0.90***
	[-0.74,-0.61]	[-0.72,-0.59]	[-0.75,-0.62]	[-0.74,-0.60]	[-0.95,-0.58]	[-0.73,-0.63]	[-0.72,-0.61]	[-1.00,-0.80]

Table S3 Heterogeneity results of mixed effect models with different interaction terms

	**Gender (Ref. = female)**		**Residence (Ref. = rural)**		**Per capita household income quantile (Ref. = 0-50%)**		**Medical insurance (Ref. = no)**		**Chronic disease (Ref. = no)**	
	Coef.	*P value*	*Coef.*	*P value*	*Coef.*	*P value*	*Coef.*	*P value*	*Coef.*	*P value*
Interactions										
**Slow increase * interaction variable**	**0.26***	**0.039**	**-0.03**	**0.811**	**0.21***	**0.047**	**0.30**	**0.122**	**-0.27***	**0.018**
Rapid * interaction variable	0.31	0.419	0.61	0.192	0.07	0.853	0.30	0.647	-0.18	0.598
Trajectory of internet usage time (Ref. = Never use)										
Slowly increase	-0.33***	0.001	-0.18	0.054	-0.30***	0.001	-0.48*	0.014	-0.14	0.077
Rapid increase	-0.46	0.091	-0.81	0.057	-0.32	0.342	-0.59	0.365	-0.27	0.221
Age	-0.01	0.165	-0.01	0.168	-0.01	0.154	-0.01	0.164	-0.01	0.172
Gender (Ref. = Female)										
Male	-1.14***	<0.001	-1.02***	<0.001	-1.02***	<0.001	-1.02***	<0.001	-1.02***	<0.001
Marital status (Ref. = No)										
Yes	-1.63***	<0.001	-1.64***	<0.001	-1.64***	<0.001	-1.64***	<0.001	-1.63***	<0.001
Education level (Ref. = Below middle school)										
High school and above	-0.57***	<0.001	-0.57***	<0.001	-0.58***	<0.001	-0.57***	<0.001	-0.57***	<0.001
Tobacco (Ref. = No)										
Yes	0.20**	0.007	0.20**	0.008	0.20**	0.008	0.20**	0.008	0.20**	0.008
Alcohol (Ref. = No)										
Yes	-0.17*	0.019	-0.17*	0.018	-0.17*	0.018	-0.17*	0.019	-0.17*	0.020
Medical insurance (Ref. = No)										
Yes	-0.45***	<0.001	-0.45***	<0.001	-0.45***	<0.001	-0.56***	<0.001	-0.45***	<0.001
Employment (Ref. = No)										
Yes	0.16**	0.010	0.17**	0.008	0.17**	0.009	0.17**	0.008	0.17**	0.008
Residence (Ref. = Rural)										
Urban	-0.67***	<0.001	-0.67***	<0.001	-0.67***	<0.001	-0.67***	<0.001	-0.67***	<0.001

Per capita household income quantile (Ref. = 0-50%)										
50-100%	-0.55***	<0.001	-0.55***	<0.001	-0.64***	<0.001	-0.55***	<0.001	-0.55***	<0.001
Chronic disease (Ref. = No)										
Yes	0.76***	<0.001	0.76***	<0.001	0.76***	<0.001	0.76***	<0.001	0.87***	<0.001
Health status	0.71***	<0.001	0.71***	<0.001	0.71***	<0.001	0.71***	<0.001	0.71***	<0.001
Life satisfaction	-0.67***	<0.001	-0.67***	<0.001	-0.67***	<0.001	-0.67***	<0.001	-0.67***	<0.001

Note: In terms of interactions, never use was the referent group, and the referent group of interaction variable was listed in the heading title of each column.

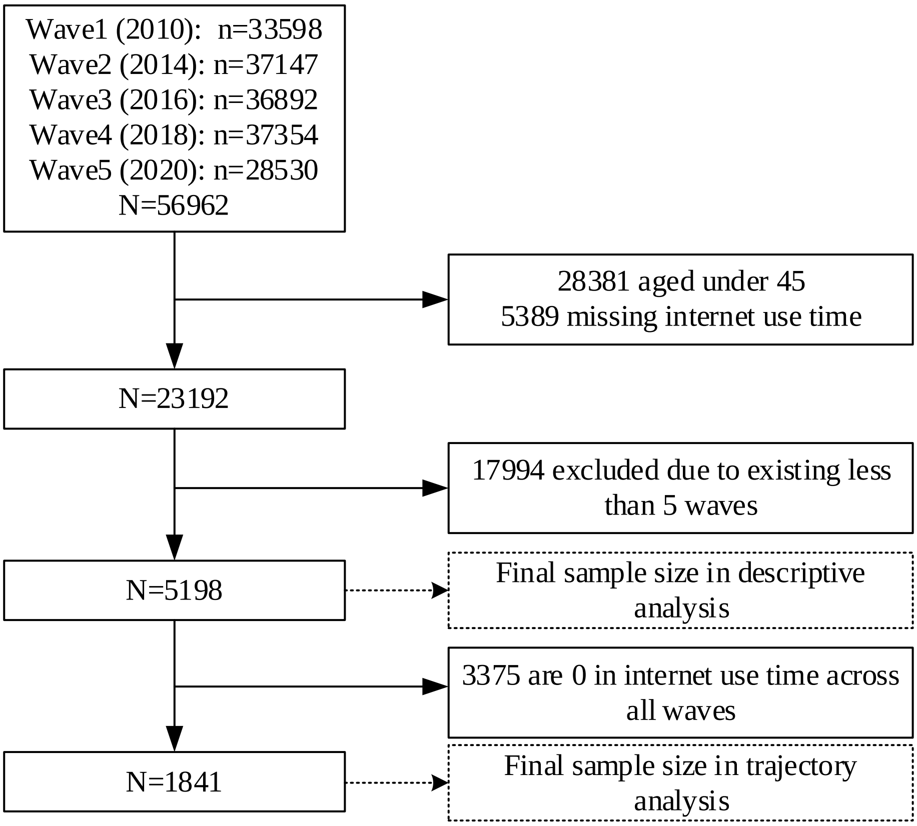

Figure S2. The flow chart of data cleaning among individuals following up 5 waves.

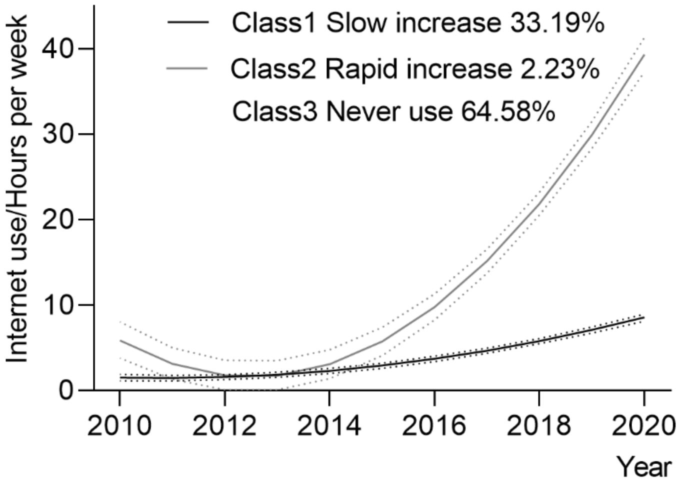

Figure S3. Internet use time trajectories among 5,198 individuals following up 5 waves

Note: The latent class mixed model identified classes of individuals with similar trajectories of internet use time over 11 years. The trajectories of internet use time were identified and showed with 95% CIs (dashed area).

Table S4. The goodness of fit indices of all classes of trajectories among individuals following up 5 waves

Latent class(es)	AIC	BIC	SABIC	each class percent (%) 1	2	3	4	5
1	61877.16	61915.79	61893.55	100				
2	**63361.64**	**63422.34**	63387.39	93.70	6.30			
3	63369.22	63451.99	63404.34	69.58	23.68	6.74		
4	62484.82	62589.67	62529.3	4.35	90.60	0	5.05	
5	62292.95	62419.87	62346.8	6.25	88.81	0	0.54	4.40

Note: AIC: Akaike Information Criterion, BIC: Bayesian Information Criterion, SABIC: Sample-Size Adjusted Bayesian Information Criterion.

Although 5 latent classes had the lowest AIC, BIC, and SABIC, however, it did not correspond with the criterion that the minimum percent of each class should be over 5%. Compared to 2 and 3 latent classes, 2 latent classes were assigned as the final result because of lower AIC, BIC, and SABIC. This result was similar to the individuals following 3 waves in the manuscript.

Table S5. Characteristics of the study population following up 5 waves in 2020

	Never use (n=3357)	Slow increase (n=1725)	Rapid increase (n=116)	*P* Value
	Mean(*SD*) / n(%)	Mean(*SD*) / n(%)	Mean(*SD*) / n(%)	
Age	66.42 (7.09)	61.9 (6.14)	62.59 (6.17)	<0.001
Gender				
Female	1745 (51.98)	782 (45.33)	64 (55.17)	
Male	1612 (48.02)	943 (54.67)	52 (44.83)	<0.001
Marital status				
No	511 (15.22)	171 (9.91)	20 (17.24)	
Yes	2846 (84.78)	1554 (90.09)	96 (82.76)	<0.001
Education level				
Below middle school	3089 (92.02)	1122 (65.04)	63 (54.31)	
High school and above	268 (7.98)	603 (34.96)	53 (45.69)	<0.001
Tobacco				
No	2401 (73.11)	1204 (70.20)	91 (78.45)	
Yes	883 (26.89)	511 (29.80)	25 (21.55)	0.030
Alcohol				
No	2777 (84.56)	1421 (82.86)	98 (84.48)	
Yes	507 (15.44)	294 (17.14)	18 (15.52)	0.293
Medical insurance				
No	274 (8.45)	98 (5.76)	6 (5.26)	
Yes	2970 (91.55)	1602 (94.24)	108 (94.74)	0.002
Employment				
No	1272 (38.00)	746 (43.32)	81 (69.83)	
Yes	2075 (62.00)	976 (56.68)	35 (30.17)	<0.001
Residence				
Rural	2037 (61.47)	631 (37.65)	16 (14.41)	
Urban	1277 (38.53)	1045 (62.35)	95 (85.59)	<0.001
Per capita household income quantile				
0-50%	2270 (69.04%)	636 (37.57%)	32 (28.32%)	
50-100%	1018 (30.96%)	1057 (62.43%)	81 (71.68%)	<0.001
Chronic diseases				
No	2337 (71.08)	1259 (73.41)	81 (69.83)	
Yes	951 (28.92)	456 (26.59)	35 (30.17)	0.195
Health status	3.39 (1.30)	3.21 (1.16)	3.34 (1.12)	<0.001
Life satisfaction	4.27 (0.90)	4.14 (0.87)	3.93 (0.93)	<0.001
Depression (CES-D score)	14.04 (4.56)	12.95 (4.13)	13.43 (4.41)	<0.001
Internet usage time	0 (0)	8.33 (7.79)	40.30 (14.62)	<0.001

Table S6. Association between trajectories of internet use time and depressive symptoms

	Depressive symptom	
	OR	95% CI
Trajectory of internet usage time (Ref. = Never use)		
Slow increase	0.87*	[0.77,0.97]
Rapid increase	0.78	[0.56,1.09]
Age	0.99	[0.99,1.00]
Gender (Ref. = Female)		
Male	0.53***	[0.47,0.60]
Marital status (Ref. = No)		
Yes	0.37***	[0.32,0.43]
Education level (Ref. = Below middle school)		
High school and above	0.66***	[0.57,0.76]
Tobacco (Ref. = No)		
Yes	1.16*	[1.01,1.32]
Alcohol (Ref. = No)		
Yes	0.80**	[0.70,0.92]
Medical insurance (Ref. = No)		
Yes	0.74***	[0.63,0.87]
Employment (Ref. = No)		
Yes	1.05	[0.94,1.18]
Residence (Ref. = Rural）		
Urban	0.64***	[0.58,0.72]
Per capita household income quantile (Ref. = 0-50%)		
50-100%	0.68***	[0.62,0.75]
Chronic disease (Ref. = No)		
Yes	1.63***	[1.48,1.80]
Health status	1.65***	[1.58,1.71]
Life satisfaction	0.64***	[0.61,0.66]

Note: Dependent variable is the binary depression symptoms. There are 26,235 observations in the mixed-effect logistic model, with “significant depressive symptoms” being 6,001 and “non-depression symptoms” being 20,234.
